# Role of Reactive Oxygen Species in Chronic Rhinosinusitis: A Narrative Review

**DOI:** 10.3390/cimb48070709

**Published:** 2026-07-11

**Authors:** Jeongmin Lee, Su Young Jung, Hye Ok Kim, Jae Min Lee, Manish Kumar Singh, Sung Soo Kim, Tong In Oh, Dong Choon Park, Seung Geun Yeo

**Affiliations:** 1 Department of Medicine, College of Medicine, Kyung Hee University, Seoul 02447, Republic of Korea; sallyljm@khu.ac.kr (J.L.); hyeokkim@khu.ac.kr (H.O.K.); 2 Department of Otorhinolaryngology-Head and Neck Surgery, Myongji Hospital, Hanyang University College of Medicine, Goyang 10475, Republic of Korea; monkiwh35@hanmail.net; 3 Clinical Research Institute, Kyung Hee University Medical Center, Seoul 02447, Republic of Korea; sunjaesa@hanmail.net; 4 Department of Otorhinolaryngology-Head and Neck Surgery, College of Medicine, Kyung Hee University Medical Center, Kyung Hee University, 23 Hoegi-dong, Dongdaemun-gu, Seoul 02447, Republic of Korea; 5 Department of Biochemistry and Molecular Biology, College of Medicine, Kyung Hee University, Seoul 02447, Republic of Koreasgskim@khu.ac.kr (S.S.K.); 6 Department of Biomedical Engineering, College of Medicine, Kyung Hee University, Seoul 02447, Republic of Korea; tioh@khu.ac.kr; 7 Department of Obstetrics and Gynecology, St. Vincent’s Hospital, College of Medicine, The Catholic University of Korea, Seoul 02447, Republic of Korea

**Keywords:** chronic rhinosinusitis, reactive oxygen species, nasal polyps, oxidative stress

## Abstract

Chronic rhinosinusitis (CRS) is an inflammatory disease of the sinonasal mucosa whose pathogenesis is characterized by complex interactions of immunological and environmental factors. The maintenance of normal sinonasal function requires a balance of sinus ostial patency, mucociliary clearance, and mucus secretion, and disruption of this balance can lead to CRS. Although many studies have examined the pathophysiology of CRS, the role of reactive oxygen species (ROS) remains incompletely understood. In this review, we analyzed 22 studies of CRS that examined the effects of ROS on epithelial barrier function, local immune responses, and tissue remodeling. The results from in vitro studies, animal models, and human tissue analyses suggest that ROS are not merely by-products of inflammation, but appear to function as key mediators in the pathophysiology of CRS, particularly in the formation and persistence of the CRS phenotype with nasal polyps (CRSwNP). In particular, CRSwNP is characterized by increased activity of enzymes in the dual oxidase (DUOX) and NADPH oxidase (NOX) families, mitochondrial dysfunction, and decreased activity of superoxide dismutase (SOD) and peroxiredoxin 2 (PRDX2). At the molecular level, these alterations increase the generation of ROS and impair antioxidant defense. At the cellular level, these alterations disrupt the epithelial barrier, activate inflammasomes, increase pyroptosis, and induce the formation of neutrophilic and eosinophilic extracellular traps. These changes culminate in the epithelial–mesenchymal transition (EMT), with the formation of nasal polyps and tissue remodeling. Increased oxidative stress can also occur in CRS without nasal polyps (CRSsNP), but this phenotype appears to have relatively preserved antioxidant defense systems, which may partly explain the more limited structural remodeling. External stimuli, such as fungal proteases, bacterial toxins, and certain antibiotics, can also increase the production of ROS and may contribute to disease chronicity. Taken together, the level and pathophysiological roles of ROS differ in the two primary phenotypes of CRS. Further mechanistic studies are needed to clarify the specific alterations of redox pathways in these two phenotypes and to develop novel therapeutic strategies that target ROS.

## 1. Introduction

Chronic rhinosinusitis (CRS) is an inflammatory disease of the nasal and paranasal sinus mucosa that has a global prevalence of more than 10% and is associated with a considerable socioeconomic burden. Patients with this disease experience symptoms such as nasal obstruction, olfactory dysfunction, facial pressure, and sleep disturbance that can persist for prolonged periods and substantially impair quality of life [1]. Traditionally, CRS was classified as a phenotype with nasal polyps (CRSwNP) or a phenotype without nasal polyps (CRSsNP). However, more recent studies consider this morphological classification as insufficient because it does not consider the heterogeneity of clinical manifestations and treatment responses. Indeed, even among patients with the same clinical diagnosis, inflammatory patterns and immune mechanisms can vary substantially. Because of this, the European Position Paper on Rhinosinusitis and Nasal Polyps (EPOS) 2020 recommended classification by the underlying endotype, particularly as type 2 inflammation (the most common type) or non-type 2 inflammation [1]. The etiological diversity of CRS is one of the major challenges in management of these patients, because recurrent disease and mucosal remodeling persists in some patients who receive standard treatments, including corticosteroid therapy and endoscopic sinus surgery. Although the introduction of biologic agents has expanded the therapeutic options, the upstream molecular mechanisms that drive early inflammatory responses and tissue remodeling in CRS remain incompletely understood [2,3].

Recent studies of CRS have examined the role of reactive oxygen species (ROS) and oxidative stress, which are caused by an imbalance of oxidants and antioxidants, in the pathophysiology of this disease. ROS are highly reactive metabolites, and include the superoxide anion (O_2_^−^), hydrogen peroxide (H_2_O_2_), and the hydroxyl radical (OH·) [4]. The body primarily generates ROS during mitochondrial oxidative phosphorylation and by enzymes in the dual oxidase (DUOX) and NADPH oxidase (NOX) families in the membranes of epithelial cells and phagocytes. Under normal physiological conditions, low levels of ROS function in metabolic ‘eustress’, in that they regulate intracellular signaling and mediate the respiratory burst of macrophages and neutrophils, responses that contribute to innate immune defense against bacterial and fungal pathogens [4]. However, an excessive level of exogenous stressors, such as air pollutants, allergens, or fungi, or endogenous stressors, such as mitochondrial dysfunction, overwhelms the antioxidant defense systems and leads to a pathological state of oxidative stress [5]. Excessive accumulation of ROS can deplete the major antioxidant enzyme systems, such as superoxide dismutase (SOD) and peroxiredoxin (PRDX), and promote the peroxidation of lipids in cellular membranes, and this can lead to the accumulation of toxic by-products, such as malondialdehyde (MDA) and 4-hydroxynonenal (4-HNE). Previous studies have established the role of oxidative injury in lower airway diseases, including asthma and chronic obstructive pulmonary disease (COPD). Consistently, recent tissue-based and transcriptomic analyses of patients with CRS have reported increased levels of oxidative stress and decreased activity of antioxidant enzymes [6].

In the sinonasal microenvironment, oxidative stress can occur as a consequence of tissue injury and can also function as a pathogenic factor that actively contributes to disease chronicity. Thus, the accumulation of ROS in the mucosa can disrupt tight junction proteins in the epithelial barrier, thereby increasing the permeability of antigens and activating NLRP3 inflammasomes in epithelial cells, culminating in programmed inflammatory cell death—pyroptosis. ROS can also promote the abnormal formation of neutrophilic and eosinophilic extracellular traps, and this can aggravate mucosal injury. The prolonged exposure to ROS can also induce the epithelial–mesenchymal transition (EMT), and this tissue remodeling can manifest as the formation of nasal polyps and stromal fibrosis [3,6]. Interestingly, ROS can act as a ‘double-edged sword’ in the setting of CRS: they can act as pathogenic mediators that stimulate epithelial and immune cells and promote inflammation and tissue remodeling; however, under certain conditions they can suppress disease by inducing the selective apoptosis of abnormally proliferating fibroblasts, thus decreasing the overall tissue burden [7]. Furthermore, the contribution of ROS to pathological and protective responses appears to be different for the CRSwNP and CRSsNP phenotypes, even though many previous studies have regarded ROS as a single toxic mediator. Integrated analyses of the cell-specific effects of ROS across the disease spectrum remain limited.

This systematic review analyzed publications that examined the in vitro molecular mechanism of CRS and recent transcriptomic studies of human tissues to comprehensively evaluate the pathological and protective effects of ROS in the development and chronicity of different types of CRS. We focused on the dual functions of ROS in different phenotypes and cell-specific redox networks, assessed the potential clinical applicability of antioxidant therapies that target specific redox nodes, and identified directions for future research.

## 2. Methods

Although numerous studies have investigated the role of oxidative stress in airway diseases, there has been no structured review that specifically focused on the molecular mechanisms of this response and the effect of ROS in the different phenotypes of CRS. Therefore, relevant studies were identified by searching five electronic databases—PubMed, SCOPUS, Cochrane Library, EMBASE, and Google Scholar—for English-language studies that were published between January 2000 and March 2026. The search used combinations of the keywords “chronic rhinosinusitis”, “nasal polyps”, “reactive oxygen species”, and “ROS”, and used Boolean operators (“AND”, “OR”) to refine the results. The predefined inclusion criteria were:

1. Original research articles;
2. Studies investigating ROS or free radical-related mechanisms in CRS; and
3. Studies that employed in vitro molecular assays, in vivo animal models, or analysis of human tissues.

The predefined exclusion criteria were:

1. Studies unrelated to CRS;
2. Studies lacking mechanistic or biochemical data regarding ROS;
3. Review articles; and
4. Studies that did not directly address redox signaling or oxidative/antioxidant imbalance.

**Figure 1:**
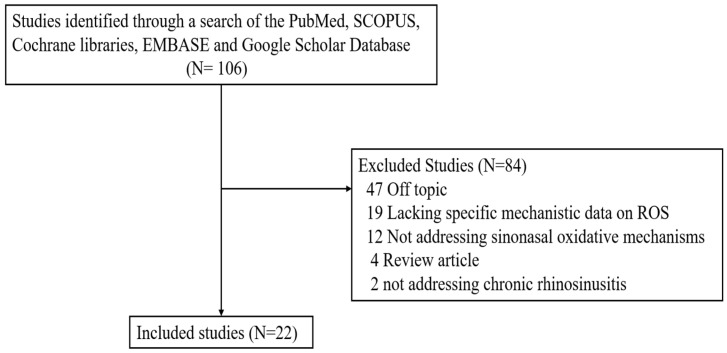
Flowchart of the literature screening and selection process.

To minimize selection bias and ensure an objective overview of the literature while maintaining a narrative synthesis, we employed a systematized literature search strategy. Comprehensive searches of the five databases were performed (Figure 1). A total of 106 studies were initially identified. Then, 47 studies that were off-topic, 19 that lacked specific mechanistic data on ROS, 4 that were review articles, 12 that did not specifically address sinonasal oxidative mechanisms, and 2 that did not address chronic sinusitis were excluded. Ultimately, 22 primary research articles were included for qualitative analysis and synthesis (Table 1). This transparent process of selecting publications was designed to minimize selection bias while maintaining the thematic focus.

## 3. Discussion

Studies of the role of ROS in the pathophysiology CRS have had inconsistent results. Early investigations suggested that ROS might not have a significant role, but more recent studies have reported that ROS functions in the development and progression of this disease. In particular, some studies reported that the level of ROS was related to the presence of nasal polyps, and other studies reported differences in the levels of ROS among control subjects, patients with CRSsNP, and patients with CRSwNP, and that many of the alterations were in the defense mechanisms of the sinonasal mucosa.

### 3.1. Association of Lipid Peroxides with the Pathogenesis of CRS

A 2002 study of CRS compared free radical-mediated damage of normal and diseased tissue samples from 13 patients undergoing functional endoscopic sinus surgery [8]. This study measured the levels of lipid peroxides (a marker of free radical-mediated lipid injury) and the LOPS/protein ratio in each sample.

The mean LOPS/protein ratio was 3.52 × 10^−5^ in normal tissue and 3.49 × 10^−5^ in diseased tissue, with no statistically significant difference. This result suggests that although free radical-mediated damage may occur in CRS, a comparison of diseased tissue and macroscopically normal tissue indicated this damage did not manifest as an increase in the level of LOPS.

However, this study had several limitations. First, although diseased and control tissues were from the same patients, the chronic nature CRS means that tissues which appeared to be macroscopically normal may have been affected by prior inflammation. In other words, the control tissues may not have been truly healthy mucosa. Second, tissue specimens were processed and frozen before analysis, and this may have induced the formation of free radicals and biomolecular damage. Indeed, previous studies have reported differences in free radical–mediated damage in frozen tissue specimens. Third, the small sample size limited the statistical power of the study [8]. It is important to note that the selection of control tissues in CRS research remains a significant challenge. While we previously noted the limitations of using macroscopically normal tissue from the same patient, we acknowledge that control tissues obtained from septoplasty or skull base surgery also exhibit anatomical and immunological variability. Future research should strive for standardized control selection criteria to better account for these potential confounding factors in redox signaling analyses.

### 3.2. Role of ROS in the Pathogenesis of CRS

#### 3.2.1. DUOX1 and DUOX2 → H_2_O_2_↑

DUOX1 and DUOX2 are the major sources of H_2_O_2_ in healthy human airways. These proteins are homologues of the NADPH oxidases (NOXs) which occur in phagocytes. Enzymes in the NOX family are membrane-bound flavoproteins that use NADPH as an electron donor to generate extracellular superoxide and H_2_O_2_. DUOX1 and DUOX2 are the major NOX enzymes in pulmonary and nasal epithelial cells, and they normally produce the H_2_O_2_ that is required by the lactoperoxidase (LPO) system, which functions in bacterial defense at the mucosal surfaces. Therefore, regulation of DUOX expression and function is an important part of the defense system of the epithelium.

A 2013 study examined normal nasal tissues from 7 subjects, nasal tissues from 6 patients with CRSwNP, and nasal tissues from 6 patients with CRSsNP that were obtained during surgery, and compared the nasal secretions of these samples [9]. This study determined the levels of cytokines and H_2_O_2_ in nasal secretions, and also measured the levels of DUOX mRNAs and DUOX proteins in these tissues. They found that the level of DUOX1 was significantly greater in CRSwNP tissues than in normal tissues and CRSsNP tissues (*p* = 0.042), and that DUOX2 expression was significantly greater in CRSwNP and CRSsNP tissues than in normal tissues (*p* = 0.008). The DUOX protein was mainly localized in the apical region of the nasal epithelium, and its expression tended to be greater in CRSwNP and CRSsNP tissues than in normal tissues. H_2_O_2_ production was approximately threefold higher in CRSwNP tissues than in normal tissues (*p* = 0.032), and the H_2_O_2_ concentration in nasal secretions had a significant positive correlation with the expression of DUOX (*p* < 0.001). The levels of four inflammatory cytokines (eotaxin, monokine induced by interferon-γ [MIG], tumor necrosis factor-α [TNF-α], and interleukin-8 [IL-8]) were also significantly greater in CRSwNP tissues than in normal tissues (*p* < 0.05), and the levels of eotaxin, MIG, and TNF-α had positive correlations with the expression of DUOX. The expression of these inflammatory cytokines was generally lower in CRSsNP tissues than in CRSwNP tissues. These findings suggest that DUOX1 and DUOX2 play important roles in the oxidative signaling that is part of the innate immune defense and in inflammatory responses of the human nasal epithelium [9].

#### 3.2.2. *Staphylococcus aureus* → Dysregulation of ROS Genes↑

The complex interactions among the host, environment, and microorganisms during the pathogenesis of CRS remain incompletely understood. Nitric oxide (NO) and ROS play important roles in innate immunity, and their production serves as a first-line defense against microbial invasion. Therefore, a 2013 study analyzed changes in the expression of genes that regulate the production of these molecules in the context of host-microbe interactions, particularly in relation to the major pathogen, *Staphylococcus aureus* [10].

This study obtained sinonasal tissue samples during surgery from 17 patients with CRS and 6 control patients undergoing surgery for pituitary macroadenoma. There were significant differences in the expression of 31 mRNAs, and these alterations were greatest in CRS patients who were biofilm-positive and polyp-positive. In particular, four mRNAs—oxidation resistance 1 (OXR1), peroxiredoxin 6 (PRDX6), neutrophil cytosolic factor 2 (NCF2), and prion protein (PRNP)—had significantly different expression among the different groups. Patients with CRS also had changes in the expression of ROS-regulatory genes, and those with S. aureus biofilms had the greatest changes. These findings may provide a mechanistic basis for the more severe clinical manifestations and poorer treatment outcomes in this patient subgroup. In addition, the presence of nasal polyps also appeared to influence the expression of mRNAs, especially in NCF2 and PRNP. These findings suggest there is a positive relationship between CRSwNP and the dysregulated production of ROS [10].

#### 3.2.3. Platycodin D → ROS↓

Pyroptosis is an inflammatory form of programmed cell death that occurs during CRS. Platycodon D (PLD) is a saponin isolated from plant roots that has demonstrated anti-inflammatory effects in various respiratory diseases. A 2024 study examined the effect of PLD on inflammatory responses and pyroptosis in CRS [11].

This study compared the nasal mucosal tissues from 21 patients with CRS and 9 control subjects who had simple septal deviation. Compared with control tissues, the CRS tissues had marked structural damage and significantly increased expression of pyroptosis-related proteins. The CRS tissues also had increased levels of ROS, decreased mitochondrial membrane potential, and structural damage of mitochondria. The in vitro pretreatment of tissues with PLD significantly inhibited these changes and activated the Nrf2/HO-1 antioxidant signaling pathway. These findings suggest that NLRP3-mediated pyroptosis plays an important role in the pathophysiology of nasal mucosal injury in CRS. Furthermore, because PLD activated the Nrf2/HO-1 pathway, decreased the production of ROS, and alleviated mitochondrial dysfunction, this compound has potential as a therapeutic to suppress inflammatory injury in the nasal epithelial cells of patients with CRS [11].

### 3.3. Role of ROS in the Pathogenesis of CRSwNP

Many diseases are closely associated with the increased oxidative stress that is caused by the excessive production of ROS and decreased production of antioxidants. Nasal polyposis is an inflammatory condition of the nasal and paranasal sinus cavities whose precise pathogenesis remains unclear. In particular, limited data are available regarding epithelial alterations in nasal polyposis and their relationship with free radical-mediated damage.

#### 3.3.1. Thioredoxin-Interacting Protein → Intracellular ROS↑

Thioredoxin (TRX) and thioredoxin-interacting protein (TXNIP) play key roles in the development of oxidative stress, although the specific mechanisms of these effects in CRSwNP remain unclear. Therefore, a 2021 study investigated the role and mechanism of TRX and TXNIP in the pathophysiology of CRSwNP by examination of 41 patients with eosinophilic CRSwNP (eCRSwNP), 43 patients with non-eosinophilic CRSwNP (neCRSwNP), and 33 control subjects [12]. This study used Western blotting, real-time PCR, and immunohistochemistry (IHC) to evaluate the expression of TXNIP and TRX in nasal tissues, used assay kits to measure the activity of malondialdehyde (MDA) and SOD in tissue homogenates, and used an ROS-detecting dye (DCFH-DA) to measure ROS production. The experiments used cultured normal human nasal epithelial cells (HNECs) and treated them with TXNIP siRNA, and other experiments examined the effect of an ROS scavenger (N-acetylcysteine, NAC).

Compared with controls, patients with CRSwNP had significantly increased levels of TXNIP and TXNIP mRNA, a greater number of TXNIP-positive cells, decreased expression of the TRX protein and TRX mRNA, increased activity of MDA, and decreased activity of SOD. The in vitro experiments also showed that treatment with TXNIP siRNA and NAC led to normalization in the levels of TXNIP, TRX, MDA, SOD, and ROS in HNECs. Taken together, these findings suggest that CRSwNP is characterized by increased expression of TXNIP, decreased expression of TRX, decreased SOD activity, and an increased level of MDA, changes that were associated with the accumulation of ROS and oxidative stress. In particular, the results indicate an imbalance of TXNIP and TRX appears to be a key factor in causing this redox balance. These alterations also occurred in HNECs, and support the importance of TXNIP-mediated regulation of oxidative stress as having an important role in the pathophysiology of CRSwNP [12].

#### 3.3.2. Imbalance of Redox-Related Genes → 4-Hydroxynonenal↑ 3-Nitrotyrosine↑

Although several studies have reported that gene products related to ROS and reactive nitrogen species functioned in the pathophysiology of CRSwNP, there has been no comprehensive analysis of oxidative stress-associated genes in this phenotype.

**Figure 2:**
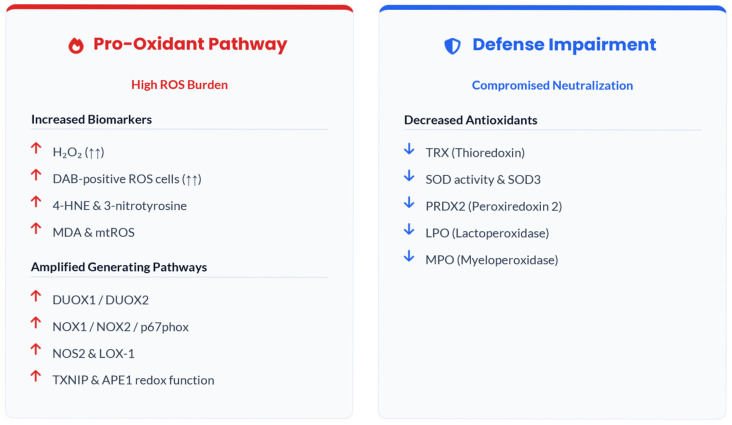
Redox Imbalance in CRSwNP. Upward arrows (↑/↑↑) indicate an increase or upregulation, and downward arrows (↓) indicate a decrease or downregulation.

Therefore, a 2022 study comprehensively analyzed the patterns in the expression of genes related to oxidative stress and antioxidant defense in 25 control subjects and 25 patients with CRSwNP. IHC analysis showed increased expression of two markers of oxidative stress (4-hydroxynonenal [4-HNE] and 3-nitrotyrosine [3-NT]) in CRSwNP nasal polyp tissues. In addition, real-time PCR analysis of 84 oxidative stress-related genes in normal nasal mucosa and nasal polyp tissues showed that 19 mRNAs were upregulated and 4 mRNAs were downregulated in the polyp tissues. The upregulated mRNAs included inducible nitric oxide synthase (iNOS) and heme oxygenase-1 (HO-1), and the down-regulated genes included LPO, myeloperoxidase (MPO), and superoxide dismutase 3 (SOD3). These findings suggest that a broad imbalance of redox regulatory networks, including the iNOS and heme peroxidase-related pathways, is closely associated with the development and progression of CRSwNP (Figure 2) [13].

#### 3.3.3. Crocin → NOS2↓ NOX1↓ NRF/HO-1↑

The clinical efficacy of antioxidant therapy for CRSwNP requires further validation. A 2024 study compared the effect of crocin (a carotenoid) on nasal polyp tissues from patients with CRSwNP and turbinate tissues from control subjects. These tissues were obtained during endoscopic surgery of the skull base or septoplasty [14]. The results showed that the expression levels of NOS2, NOX1, HO-1, and SOD2 were greater in the nasal epithelial cells and macrophages derived from nasal polyp tissues. In addition, the levels of two oxidases (NOS2 and NOX1) showed positive correlations with two pro-inflammatory cytokines (IL-5 and IL-6), whereas the expression of two antioxidant enzymes (HO-1 and SOD2) showed negative correlations with two pro-inflammatory cytokines (IL-13 and IFN-γ). The in vitro application of crocin inhibited the differentiation into M1 and M2 macrophages, decreased the expression of NOS2 and NOX1, and increased the antioxidant capacity of M2 macrophages. Crocin also activated the anti-oxidant KEAP1/NRF2/HO-1 signaling pathway in human nasal epithelial cells after stimulation by staphylococcal enterotoxin B (SEB) or lipopolysaccharide (LPS). These antioxidant and anti-inflammatory effects also occurred in sinonasal tissue explants.

Taken together, these findings suggest that oxidative stress plays an important role in the development and progression of CRSwNP by amplifying various inflammatory responses, and that antioxidant compounds such as crocin have potential as therapeutics [14].

#### 3.3.4. LOX-1 → ROS- and NO-Related Inflammation↑

Scavenger receptors (SRs) are a family of transmembrane receptors that have protective effects in a wide range of host-environment interactions. Some SRs, such as LOX-1, normally produce ROS and others, such as SR-B1, normally act as antioxidants. Therefore, a 2020 study evaluated the relationship of these two major SRs with different clinical phenotypes and histologic subtypes of CRS.

This study examined ethmoid sinus mucosal tissues and blood samples from patients with CRSwNP (*n* = 31), patients with CRSsNP (*n* = 13), and control subjects (*n* = 19). It used RT-PCR to measure the expression of the LOX-1 and SR-B1 mRNAs, and ELISA and IHC to measure the expression and distribution of the LOX-1 and SR-B1 proteins. LOX-1 expression was significantly increased in the CRSwNP group compared with the control group. In contrast, SR-B1 expression did not differ significantly among the three groups. In addition, LOX-1 mRNA expression levels showed a significant positive correlation with sinus CT scores. The serum level of LOX-1 did not differ among the groups; however, the LOX-1 level in sinus tissues was significantly greater in the CRSwNP group than in the controls. Immunostaining showed that LOX-1 was mainly expressed in inflammatory cells and vascular endothelial cells. These findings suggest that increased expression of LOX-1 may contribute to the pathophysiology of CRSwNP by promoting the generation of ROS and enhancing crosstalk with nitric oxide (NO) [15].

#### 3.3.5. NOX2 → ROS↑ → NLRP3 Inflammasomes↑

A study from 2024 used single-cell RNA sequencing data from 40 patients with CRSwNP and 20 patients with CRSsNP to analyze oxidative stress-related mechanisms in the epithelial cells of patients with these diseases [16]. The analysis showed that scores for oxidative stress and NLRP3 inflammasome activation were significantly greater in the epithelial cells of patients with CRSwNP.

In particular, CRSwNP epithelial cells had increased expression of NOX2, increased generation of ROS, increased activation of NLRP3 inflammasomes, and increased expression of epithelial alarmin. These findings suggest that NOX2 may act as a key regulatory factor that activates the NLRP3 inflammasome by generation of ROS, thereby promoting inflammatory responses and disease progression. Furthermore, because the ROS-NLRP3 inflammasome axis is important in the pathogenesis of other chronic inflammatory diseases, modulation of oxidative stress by targeting NOX2 may be a promising therapeutic strategy for treatment of chronic inflammatory diseases [16].

#### 3.3.6. NOX and p67phox → 4-HNE↑

Recent studies have suggested that enzymes in the NOX family and oxidative stress may play important roles in the pathophysiology of CRSwNP. Therefore, a 2020 study aimed to clarify the role of NADPH oxidase in the pathophysiology of CRSwNP by measuring the expression of different NADPH oxidase subunits and a marker of oxidative stress (4-hydroxynonenal [4-HNE]) in nasal polyp tissues and normal nasal mucosa.

This study examined 13 patients with CRSwNP and 9 normal subjects and measured the expression of four NADPH oxidase subunits (gp91phox, p67phox, p47phox, and p22phox) using Western blotting and real-time PCR. It also used IHC and immunofluorescence staining to assess the levels of p67phox and 4-HNE in nasal polyp tissues and normal nasal mucosa. Western blotting and real-time PCR analyses showed that the mRNA and protein levels of p67phox were significantly greater in nasal polyp tissues than in control mucosa (*p* = 0.004). Immunostaining also demonstrated expression of p67phox in the eosinophils and neutrophils of nasal polyp tissues, but not in macrophages. In addition, the level of 4-HNE was significantly greater in nasal polyp tissues than in control mucosa (*p* = 0.001). These findings suggest that increased p67phox expression and the associated increased oxidative stress may play an important role in the pathophysiology of CRSwNP [17].

#### 3.3.7. Mitochondrial Dysfunction → mtROS↑

CRSwNP is closely associated with mitochondrial dysfunction, structural alterations of mitochondria, and the production of ROS. Therefore, a 2020 study evaluated functional and morphological changes in the mitochondria of epithelial cells from nasal polyps and nasal epithelial cells stimulated with S. aureus enterotoxin B (SEB) [18]. The comparison of 30 patients with CRSwNP and 15 healthy controls demonstrated that the expression of oxidative phosphorylation complexes was significantly greater in nasal polyp tissues from patients with CRSwNP than in uncinate tissues (UTs) from control subjects. The in vitro application of SEB stimulated the production of mitochondrial ROS (mtROS), and MitoTEMPO (an mtROS scavenger) significantly decreased this effect. At the tissue level, the concentration of mtROS was significantly greater in the nasal polyps of patients with CRSwNP than in the UTs from controls and the UTs from patients with CRSwNP. Disruption of mitochondrial structure and the expression of fission- and fusion-related proteins, such as mitofusin and optic atrophy protein, were significantly greater in the nasal polyps of patients with CRSwNP than in the UTs from controls and the UTs from patients with CRSwNP. These findings indicate that increased production of mtROS, mitochondrial dysfunction, and structural alterations of mitochondria occur in the nasal epithelial cells of patients with CRSwNP, and these may play an important role in the pathophysiology of this disease [18].

#### 3.3.8. H_2_O_2_ → RV16-Induced Responses↓

Abnormalities in innate immune defense mechanisms, including an oxidant-antioxidant imbalance, play an important role in the pathophysiology of CRS. Thus, a 2023 study [19] examined the effect of attenuation of oxidative stress on the secretion of antiviral interferons in the human sinonasal mucosa. The study population consisted of 49 patients with CRS (23 with CRSsNP and 26 with CRSwNP) and 65 control subjects (15 patients with blowout fractures and 50 patients with septal deviation or hypertrophic rhinitis).

The results showed that addition of rhinovirus 16 (RV16) infection or poly I:C (a ‘viral mimic’) increased the expression of a type I interferon (IFN-β), two type III interferons (IFN-λ1 and IFN-λ2), and multiple interferon-stimulated genes (ISGs). However, pretreatment with H_2_O_2_ significantly attenuated the upregulation of these interferons and ISGs. In contrast, treatment with an antioxidant (NAC) modulated this effect. Consistently, H2O2 pretreatment also decreased the expression of TLR3, RIG-I, MDA5, and IRF3, but NAC treatment modulated this effect. Cells treated with Nrf2 siRNA had decreased secretion of antiviral interferons, whereas treatment with sulforaphane (an Nrf2 activator) increased the interferon secretory capacity. These findings suggest that RV16-induced antiviral interferon responses can be suppressed by oxidative stress, and that the Nrf2-mediated antioxidant pathway plays an important role in regulating this immune response [19].

#### 3.3.9. *Aspergillus fumigatus* → NADPH Oxidase-Derived ROS and mtROS↑

CRS is characterized by the infiltration of inflammatory cells into the sinonasal mucosa, and the formation of eosinophilic extracellular traps (EETs) and neutrophilic extracellular traps (NETs) are part of the immune response. A 2023 study investigated the effects of two airborne fungi—*Alternaria alternata* and *Aspergillus fumigatus*—on the formation of EETs and NETs [29].

This study isolated nasal epithelial cells, eosinophils, and neutrophils from 14 patients with eosinophilic CRS (eCRS), 14 patients with non-eosinophilic CRS (neCRS), and 5 healthy controls. After fungal stimulation, these researchers evaluated transepithelial migration of eosinophils and neutrophils, the release of EETs and NETs using SYTOX Green staining, and the effect of ROS inhibitors to assess the role of ROS production in the formation of extracellular traps. The results indicated that the numbers of EETs and NETs were significantly greater in patients with eCRS than with neCRS. A. fumigatus significantly increased the formation of EETs and NETs, and this effect was more pronounced in eCRS. In patients with CRS, eosinophils and neutrophils suppressed the metabolic activity of A. fumigatus, possibly because EETs and NETs have fungicidal effects. EET formation was associated with increased intracellular activity of NADPH oxidase; NET formation was associated with increased activity of NADPH oxidase and an increased level of mtROS. These findings suggest that ROS mediate the formation of granulocytic extracellular traps, particularly in eCRS, and may contribute to the increased mucus viscoelasticity, epithelial cytotoxicity, tissue damage, and persistent inflammatory responses in these patients [29].

#### 3.3.10. APE1 Redox Function → ROS↑

Apurinic/apyrimidinic endonuclease 1 (APE1) is a multifunctional protein that functions in DNA repair and as a redox regulator (Figure 2). Its redox function regulates various transcription factors that function in cell survival, angiogenesis, and inflammatory responses. Therefore, a 2025 study examined the role of APE1 redox function in the pathogenesis of CRS and the therapeutic potential of targeting APE1 using redox inhibitors [21]. This study measured APE1 expression and function in nasal polyp tissues from 30 patients with CRSwNP and nasal tissues from 30 healthy controls. The results indicated that APE1 had greater expression in patients with CRSwNP, and that this increase was closely associated with eosinophil infiltration, mucus hypersecretion, epithelial damage, and oxidative stress. Treatment with C10 (a small-molecule selective inhibitor of APE1 redox activity) markedly decreased the production of IL-25 in tuft cells, and also decreased the levels of type 2 cytokines, including IL-4, IL-5, and IL-13, and the infiltration of Th2 cells and innate lymphoid cells. APE1 inhibition decreased the production of ROS, the imbalance of oxidants and antioxidants, and mitochondrial damage. These findings suggest that the redox function of APE1 plays an important role in the pathophysiology of CRS by increasing type 2 inflammation, mitochondrial oxidative stress, mucus production, and dysfunction of the epithelial barrier. Furthermore, C10 may have potential as a therapeutic that suppresses type 2 inflammation and ameliorates the pathogenesis of CRS [21].

#### 3.3.11. PRDX2 → H_2_O_2_ and ROS↓

To elucidate the role of peroxiredoxin 2 (PRDX2) in the pathophysiology of CRSwNP, a 2026 study compared nasal tissues using a discovery cohort consisting of 12 patients with CRSwNP and 13 healthy controls, and a validation cohort consisting of 30 patients with CRSwNP (15 patients with eCRSwNP and 15 with neCRSwNP) and 20 healthy controls [22]. As expected, patients with CRSwNP had a greater level of ROS, particularly in the nasal epithelium. The expression of PRDX2 had a positive correlation with E-cadherin (an epithelial marker) but a negative correlation with EMT markers (TGF-β1 and vimentin). The in vitro stimulation with H_2_O_2_ increased the production of ROS and promoted EMT in nasal epithelial cells (NECs). In contrast, PRDX2 overexpression significantly inhibited the H_2_O_2_-induced increases in ROS, EMT-related changes, and the H_2_O_2_-induced activation of the TGF-β1/SMAD signaling pathway (a key regulator of EMT). These findings suggest that PRDX2 regulates ROS-induced epithelial remodeling and may influence disease progression in CRSwNP by targeting the TGF-β1/SMAD pathway, and suggest that the overproduction of ROS has an important role in the pathophysiology of CRSwNP [22]. Furthermore, as highlighted in a recent comprehensive review by Tai et al. [30], the dysregulation of the body’s intrinsic antioxidant defense systems, particularly the impaired profiles of superoxide dismutase (SOD) enzymes and peroxiredoxins, is a fundamental pathogenic factor in CRSwNP. Their analysis demonstrates that the severe imbalance between reactive oxygen species (ROS) composed of free radicals and these specific antioxidant enzymes significantly contributes to the persistent pathology of nasal polyps. This firmly supports our findings that compromised neutralization mechanisms and sustained oxidative stress are central to the chronicity and structural remodeling observed in the CRSwNP phenotype.

#### 3.3.12. Bleomycin A5 → ROS↑

CRSwNP is characterized by the infiltration of fibroblasts and the subsequent accumulation of extracellular matrix (ECM). Previous studies suggested that intralesional injection of bleomycin A5 (BLE-A5), a glycopeptide antibiotic that induces DNA damage, was an effective and safe treatment for CRSwNP, although the underlying mechanism remains unclear. Therefore, a 2019 study investigated the mechanism of BLE-A5-induced apoptosis in nasal polyp-derived fibroblasts (NPDFs) [23].

This study obtained nasal polyp tissues from 6 patients undergoing functional endoscopic sinus surgery and normal mucosa (NM) from the inferior turbinate of 6 other patients (controls) who were undergoing surgery for nasal septal deviation. A comparison of NPDFs and normal mucosa-derived fibroblasts (NMFCs) indicated the NPDFs had higher proliferative capacity and a higher basal level of ROS. Treatment with BLE-A5 increased the production of ROS more in the NPDFs than in the NMFCs during the early phase, and the antioxidant glutathione (GSH) modulated this effect. These findings suggest that the basal level of ROS in fibroblasts is an important determinant of sensitivity to BLE-A5, and that additional accumulation of ROS is essential for achieving BLE-A5-induced apoptosis [23].

### 3.4. Differences in ROS Levels and Antioxidant Responses Between CRSwNP and CRSsNP

#### 3.4.1. Secondhand Smoke → ROS↑

A 2013 study assessed the presence of ROS in the sinus tissues of patients with CRS and evaluated the effect of secondhand smoke exposure, determined by hair nicotine levels, on ROS production [23]. This study obtained sinus tissue samples from 69 patients undergoing sinus surgery and measured ROS using DAB staining. Among patients without exposure to SHS, the number of DAB-positive cells per high-powered field (hpf) was greatest in the CRSwNP group, followed by the CRSsNP group, and then the control group. The number of DAB-positive cells per hpf was significantly higher in controls exposed to SHS than in unexposed controls. However, exposure to SHS did not significantly affect DAB staining in patients with CRSsNP or CRSwNP. These findings suggest that ROS expression differs in patients with different CRS phenotypes, and that exposure to SHS appears to increase the level of ROS in normal sinus mucosa, but not in the mucosa of patients with CRS [24].

#### 3.4.2. Redox-Related DEGs → Oxidative Stress↑

A 2025 study used a systematic approach to characterize the redox-related gene expression profile in CRSsNP, the association of this profile with oxidative damage, and the differences between CRSsNP and CRSwNP tissues. Nasal mucosal tissues were collected from 24 patients with CRSsNP and 24 control subjects. Of these, samples from 6 controls and 6 patients with CRSsNP were used for the initial 84-gene real-time PCR microarray analysis, while an additional independent set of 18 controls and 18 patients with CRSsNP was used for customized real-time PCR microarray validation. There were 27 differentially expressed genes (DEGs) in CRSsNP (24 upregulated and 3 downregulated) relative to the control; AKR1C2, GCLM, GPX2, NOS2, and NQO1 were upregulated more than fourfold, and LPO was downregulated more than fourfold. These changes in gene expression were closely associated with increased oxidative stress in the CRSsNP nasal mucosa. In addition, comparison of the 27 DEGs in this study with the 23 previously reported DEGs in CRSwNP indicated there were 16 unique redox-related DEGs of these two phenotypes. STRING-based protein–protein interaction network analysis showed that CRSsNP tissues had an ‘adaptive antioxidant defense’ signature and that CRSwNP was dominated by a ‘pro-inflammatory and oxidant pathway’ signature [25].

### 3.5. ROS Weakens the Defense Mechanisms of the Sinuses and Contributes to Disease Chronicity

Specific fungal proteases, such as serine proteases derived from *Alternaria alternata*, have been shown to directly trigger intracellular ROS bursts, which subsequently downregulate critical tight junction proteins and cause severe epithelial barrier dysfunction [31]. Similarly, bacterial exotoxins—most notably *Staphylococcus aureus* alpha-toxin (Hla)—trigger massive ATP release and calcium influxes in airway epithelial cells; these act as autocrine/paracrine inflammatory signals that disrupt mitochondrial membrane potential, ultimately leading to excessive mitochondrial ROS (mtROS) production [32]. Even therapeutic interventions can exacerbate this oxidative burden; recent mechanistic and chemical proteomics studies demonstrate that bactericidal antibiotics, particularly fluoroquinolones, induce collateral oxidative damage in mammalian mucosal cells by disrupting mitochondrial respiration through multiple off-target interactions and the inhibition of mitochondrial topoisomerase 2, ultimately triggering significant superoxide accumulation [33,34].

#### 3.5.1. *Alternaria alternata* → ROS Generation↑

*A. alternata* is a fungus commonly detected in nasal secretions and plays an important role in the pathophysiology of airway diseases. Therefore, a 2019 study investigated the effects of Alternaria on the junction complex of nasal epithelial cells. These researchers isolated HNECs from the inferior turbinate mucosa of 10 patients undergoing septal surgery and cultured these cells under air-liquid interface (ALI) conditions, followed by stimulation with *A. alternata*. This treatment increased the intracellular production of ROS and decreased transepithelial resistance (TER). It also decreased the expression of the mRNAs and proteins of zonula occludens-1, occludin, and claudin-1 (tight junction proteins), but had no effect on adherens junction proteins. These findings suggest that Alternaria-induced ROS may play an important role in disrupting the nasal epithelial barrier and amplifying inflammatory responses in the nasal mucosa [20].

#### 3.5.2. SOD → Aspergillus- and Alternaria-Induced IL-6/IL-8 Production↓

A 2015 study isolated human sinonasal epithelial cells (HSNECs) from 7 control subjects and 9 patients with CRSwNP, stimulated these cells with A. fumigatus or *A. alternata*, and then treated them with SOD. Each fungus increased the production of IL-6 and IL-8 in the HSNECs from control subjects and patients with CRSwNP. However, SOD had no anti-inflammatory effect in control cells, but did have an anti-inflammatory effect in HSNECs from patients with CRSwNP. In particular, SOD significantly decreased the production of IL-6 and IL-8 after Alternaria stimulation and significantly decreased the production of IL-8 after Aspergillus stimulation. Although this study did not perform direct measurements of ROS, the attenuation of fungal antigen-induced cytokine responses by SOD supports the interpretation that ROS-related inflammatory signaling has a role in the response to fungi by CRSwNP epithelial cells. Therefore, together with the previous findings of Alternaria-induced ROS-dysfunction of the epithelial barrier, these findings provide complementary evidence that fungal exposure may amplify oxidative stress and inflammatory responses in CRSwNP nasal epithelia [26].

#### 3.5.3. Sulforaphane → ROS↓ → Levofloxacin-Induced Apoptosis↓

Antibiotics are widely used to treat patients who have CRS due to infection. Previous studies found that bactericidal antibiotics can induce the production of ROS, inflammatory responses, and cell death in cultured human sinonasal epithelial cells (SNECs). Sulforaphane is a potent activator of the antioxidant Nrf2 signaling pathway. Therefore, a 2016 study examined the relationship between levofloxacin-induced ROS generation and cell death, and the effects of sulforaphane [27].

This study examined 10 tissue samples, 7 from control patients (without CRS) and 9 from patients with CRS. The control group consisted of patients undergoing dacryocystorhinostomy, cerebrospinal fluid leak repair, or endoscopic skull base surgery. The researchers collected mucosal tissues from the ethmoid sinus and cultured them under ALI conditions using standard methods. Levofloxacin significantly increased the activity of the pro-apoptotic enzyme caspase-3 by 5.9-fold (*p* = 0.01); pretreatment with sulforaphane led to an increase of only 1.9-fold. Levofloxacin also increased the level of ROS by 1.2- to 1.8-fold; pretreatment with sulforaphane led to an increase of only 1.0- to 1.3-fold (although this difference was not statistically significant). These findings suggest that levofloxacin may induce an apoptotic response due to the increased production of ROS, and that sulforaphane may partially suppress this effect by increasing Nrf2 activity [27].

#### 3.5.4. Amoxicillin and Levofloxacin → ROS↑ LDH↑

A 2017 study investigated the effect of bactericidal antibiotics on the induction of ROS and epithelial injury in human sinonasal epithelial cells (SNECs) [28]. Bactericidal antibiotics can induce the formation of ROS in mammalian cells by increasing mitochondrial dysfunction, and long-term use can lead to oxidative tissue damage and other adverse consequences. However, the exact nature of the relationship between antibiotic exposure and ROS generation in sinonasal epithelial cells is uncertain.

To address this issue, these researchers obtained ethmoid sinus mucosal tissues from 13 control subjects without CRS who were undergoing dacryocystorhinostomy, cerebrospinal fluid leak repair, or endoscopic skull base surgery. The tissues were cultured under ALI conditions to generate differentiated SNECs, and the differentiated SNECs were exposed to bactericidal antibiotics (amoxicillin or levofloxacin) or a bacteriostatic antibiotic (clarithromycin) for 24 h. Amoxicillin and levofloxacin led to increased production of ROS and secretion of LDH (*p* < 0.05), and this increased level of ROS was significantly associated with increased expression of Nrf2-mediated antioxidant genes and of two pro-inflammatory cytokines, TNF-α and IL-1β (*p* < 0.05). In contrast, clarithromycin did not cause significant changes in the production of ROS or the expression of these pro-inflammatory cytokines. These findings demonstrate that bactericidal antibiotics can increase ROS generation in SNECs, and that this is accompanied by activation of inflammatory and antioxidant responses and injury of epithelial cells. This suggests that long-term or inappropriate use of antibiotics for treatment of sinusitis may lead to oxidative damage of tissues in the sinonasal epithelium [28].

### 3.6. Limitations of the Current Literature and Future Directions

A limitation of the current literature is the significant methodological heterogeneity in how ROS and oxidative stress are quantified. Across the analyzed studies, detection methods ranged from immunohistochemistry for damage markers (e.g., 4-HNE, 3-nitrotyrosine) and fluorescent ROS probes (e.g., DCFH-DA) to transcriptomic profiling of redox enzymes. This diversity in experimental models and measurement units precludes the statistical pooling of data for a formal meta-analysis. Future research should aim to establish standardized protocols for quantifying oxidative stress in sinonasal tissues, which would facilitate rigorous quantitative comparisons and meta-analyses.

## 4. Conclusions

We assessed the pathological and protective effects of ROS on the pathogenesis and chronicity of CRS by analyzing 22 studies that were identified by a systematic search of the literature using predefined search criteria. The evidence indicates that ROS are not merely by-products of inflammation during the pathogenesis of CRS; instead, ROS are key regulatory molecules that can contribute to disease initiation and persistence.

**Figure 3:**
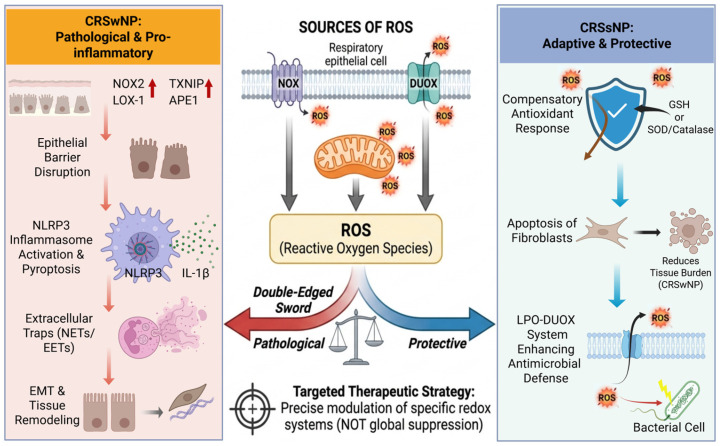
Schematic representation of the ‘double-edged sword’ role of ROS in CRS pathophysiology. ROS generated from intracellular and extracellular sources drive divergent cellular responses depending on the CRS phenotype. In CRSwNP (**left**), ROS accumulation and upregulation of specific targets trigger pathological pathways, including barrier disruption, NLRP3 inflammasome activation, extracellular trap formation, and EMT, resulting in inflammation and fibrosis. Conversely, in CRSsNP (**right**), ROS can mediate a compensatory adaptive antioxidant response, inducing fibroblast apoptosis and enhancing antimicrobial defense. This highlights the need for targeted therapeutic strategies focused on precise modulation rather than global ROS suppression. The red upward arrow indicates an increase or upregulation, and the large downward arrow represents the downstream signaling pathway. Created in BioRender. Taehyeok, S. (2026) BioRender.com/24d79of.

ROS are generated from multiple intracellular and extracellular sources, including DUOX, NOX, and mitochondrial enzymes, and they mediate various pathological processes in CRS, such as disruption of the epithelial barrier, activation of NLRP3 inflammasomes, pyroptosis, formation of extracellular traps, and induction of the EMT. In particular, studies of CRSwNP indicate that this phenotype is characterized by increased expression of DUOX, NOX2, TXNIP, LOX-1, and that the increased APE1 expression in this phenotype is associated with increased generation of ROS and is linked to type 2 inflammation, mucus hypersecretion, tissue remodeling, and fibrosis. In contrast, CRSsNP appears to be characterized by a more compensatory adaptive antioxidant response. This suggests that different CRS phenotypes may arise under similar oxidative conditions and that ROS do not always function solely as pathological mediators in CRS. Under certain conditions, ROS may exert protective effects by inducing the apoptosis of fibroblasts or enhancing antimicrobial defense through the LPO–DUOX system. Thus, ROS appears to function as a ‘double-edged sword’ in CRS. This suggests that therapeutic strategies should not simply aim for the global suppression of ROS, but should instead focus on more precise modulation of specific redox systems in specific cells and specific signaling pathways (Figure 2 and Figure 3).

Indeed, novel targeted therapeutic strategies should focus on the precise modulation of specific redox systems rather than non-specific global ROS suppression. Specifically, these strategies include the pharmacological activation of antioxidant pathways using Nrf2 activators (e.g., sulforaphane, Platycodon D) or KEAP1-NRF2/TXNIP-TRX modulators (e.g., crocin). Furthermore, targeted suppression of ROS overproduction can be achieved through inhibitors of ROS-generating enzymes, such as NOX, DUOX, LOX-1, and specific APE1 redox inhibitors like C10. Additionally, mitochondrial-targeted therapies utilizing mtROS scavengers (e.g., Mito-TEMPO) offer a distinct approach to restoring mitochondrial function. Conversely, exploiting the “double-edged sword” nature of ROS via selective pro-oxidant interventions, such as utilizing bleomycin A5, can beneficially induce ROS-mediated apoptosis in over-proliferating fibroblasts. These pathway-specific approaches hold significant potential as new therapeutics for refractory CRS.

## Figures and Tables

**Table 1:** Studies that examined the role of ROS in CRS.

Author/ Year/ Reference	Study Design	Sample	Detection Method	Target Substance(s) Associated with ROS	Results and Authors’ Conclusions
Friedman et al., 2002 [8]	Human; paired tissue comparison (pilot pathophysiologic study)	13 CRS patients undergoing FESS; diseased mucosa vs. healthy-appearing mucosa from same patient	Lipid peroxide (LPO) assay; protein quantification; LPO/protein ratio comparison	Free radical-mediated LPO	Pilot study of 13 paired samples from CRS patients indicated the LOPS/protein ratio was similar for healthy-appearing mucosa and diseased mucosa (healthy: 3.52 × 10^−5^, diseased: 3.49 × 10^−5^, 95% CI: −3.00 × 10^−5^ to 2.94 × 10^−5^). Conclusion: Free radical-induced damage, as indicated by the LOPS/protein ratio, was the same in CRS and control tissues.
Cho et al., 2013 [9]	Human; cross-sectional analysis of tissue and secretions	Normal (*n* = 7), CRSwNP (*n* = 6), CRSsNP (*n* = 6) surgical patients	qRT-PCR and protein localization for DUOX1/2; H2O2 in nasal secretions; cytokine multiplex (Luminex)	DUOX1/DUOX2; H2O2; eotaxin, MIG, TNF-α, IL-8	The level of *DUOX1* was significantly greater in CRSwNP tissues than in normal and CRSsNP tissues (*p* = 0.042). The level of *DUOX2* was greater in CRSwNP and CRSsNP tissues than in normal tissues (*p* = 0.008). Nasal secretion and H_2_O_2_ production were greater in CRSwNP and CRSsNP tissues than in normal tissues (*p* = 0.032), and correlated with *DUOX* expression (*p* < 0.001). The levels of eotaxin, MIG, TNF-α, and IL-8 were greater in CRSwNP tissues than normal tissues (*p* < 0.05), and the levels of eotaxin, MIG, and TNF-α correlated with the level of *DUOX*. Conclusion: DUOX1 and DUOX2 were upregulated in CRS, there was a close link between *DUOX* expression and H_2_O_2_ release, and there were correlations between key cytokines and *DUOX* expression. DUOX may have an important role in the inflammatory response of CRS.
Jardeleza et al., 2013 [10]	Human; gene-expression profiling by phenotype/endotype	Sinonasal tissue from CRS vs. controls; subgrouped by *S. aureus* biofilm (B+/B−) and polyp status (P+/P−)	PCR array (84 genes) related to NO/ROS regulation; biofilm detection by FISH	OXR1, PRDX6, NCF2, PRNP and other NO/ROS regulatory genes	An 84-gene PCR array of genes related to production of NO and ROS found changes in the levels of 31 genes in CRS, with the largest changes in patients who were *S. aureus* biofilm-positive and polyp-positive (B+P+). A comparison of B+P+ and control patients indicated several specific differences: *NCF2* (+2.8-fold, *p* = 0.03), *PRDX6* (−2.7-fold, *p* = 0.003), and *OXR1* (−4.3-fold, *p* = 0.0001). Conclusion: Bacterial biofilm and nasal polyps affected the expression of NO/ROS-related genes, consistent with altered innate immune responses in CRS.
Wang et al., 2024 [11]	Human tissue + in vitro CRS-like model	CRS nasal mucosa vs. septal deviation controls; cultured HNEpCs stimulated with LPS/ATP ± platycodon D	H&E and IF for pyroptosis markers; EthD-I/TUNEL/TEM; WB for NLRP3, GSDMD-NT, IL-1β/IL-18; ROS (H2DCFDA); JC-1 mitochondrial potential; Nrf2/HO-1 pathway	NLRP3 inflammasome-mediated pyroptosis; ROS/mitochondrial damage; Nrf2/HO-1 activation by PLD	CRS nasal mucosa had higher levels of pyroptosis markers than controls (GSDMD-NT, *p* < 0.05; NLRP3 and cleaved caspase-1, *p* < 0.01). In LPS/ATP-stimulated HNEpCs, PLD (2.5–5.0 μM) ameliorated cell injury and decreased the level of LDH (*p* < 0.01), suppressed activation of pyroptosis and inflammasomes (EthD-I, TUNEL assay, GSDMD-NT, *p* < 0.01; NLRP3/ASC/cleaved caspase-1, *p* < 0.05), decreased the levels of inflammatory cytokines (IL-18, *p* < 0.01; IL-1β, *p* < 0.05; extracellular release, *p* < 0.01), decreased oxidative stress and mitochondrial dysfunction (ROS and JC-1 recovery, *p* < 0.01), and activated Nrf2/HO-1 (*p* < 0.01). An Nrf2 inhibitor (ML385) blunted these effects (*p* < 0.01). Conclusion: NLRP3-mediated pyroptosis contributed to mucosal impairment in CRS. PLD inhibited NLRP3-mediated pyroptosis via activation of Nrf2/HO-1, decreased the production of ROS, and mitigated mitochondrial damage.
Lin et al., 2021 [12]	Human tissue + in vitro knockdown study	CRSwNP vs. controls; cultured human nasal epithelial cells with TXNIP siRNA ± NAC	WB/qPCR/IHC for TXNIP/TRX; tissue MDA and SOD activity assays; cellular ROS probe (DCFH-DA)	TXNIP–TRX axis; ROS; MDA; SOD; NAC scavenging	The levels of TXNIP and TRX had a negative correlation in CRSwNP tissues. TXNIP had a positive correlation with MDA (r = 0.663, *p* < 0.001) and a negative correlation with SOD (r = −0.715, *p* < 0.001). TXNIP knockdown in ALI HNECs decreased oxidative-stress and increased antioxidant activity (*p* < 0.05). Conclusion: TXNIP upregulation promoted oxidative stress in the nasal epithelium and may contribute to the pathogenesis of CRSwNP.
Tsai et al., 2022 [13]	Human; PCR-array transcriptomics + validation	Controls (*n* = 25) vs. CRSwNP (*n* = 25) nasal mucosa/NP tissues	IHC for 4-HNE and 3-nitrotyrosine; 84-gene oxidative stress PCR array; RT-PCR/WB validation; ROC analysis	Redox genes (iNOS, HO-1 up; LPO, MPO, SOD3 down); oxidative damage markers 4-HNE/3-NT	CRSwNP nasal polyp tissues had increased levels of oxidative-damage markers (4-HNE, 3-nitrotyrosine) and differential expression of oxidative-stress genes: NOS2/iNOS (919.38-fold increase, *p* = 0.037) HMOX1/HO-1 (4.00-fold increase, *p* = 0.00021), LPO (17.29-fold decrease, *p* = 0.00476), MPO (10.34-fold decrease, *p* = 0.04083), and SOD3 (2.89-fold decrease, *p* = 0.03504). ROC analysis suggested that LPO (AUC: 0.947), MPO (AUC: 0.907), SOD3 (AUC: 0.883), NOS2 (AUC: 0.853), and HMOX1 (AUC: 0.858) have potential as predictors or endotype markers for CRSwNP. Conclusion: CRSsNP possesses “an adaptive antioxidant defense signature”, while CRSwNP tends to exhibit “a pro-inflammatory/oxidant pathway”, highlighting the unique redox gene sets and pathway differences between CRSsNP and -wNP.
Zhou et al., 2024 [14]	Human tissue + in vitro/ex vivo intervention	Nasal polyp tissues; cultured HNEpCs and primary macrophages; nasal explants; crocin treatment	IHC/IF/WB/qPCR for NOS2, NOX1, HO-1, SOD2; correlation with cytokines; macrophage polarization assays; KEAP1/NRF2/HO-1 pathway readouts	Oxidases (NOS2/NOX1) and antioxidants (HO-1/SOD2); crocin; NRF2 pathway	CRSwNP tissues and derived cells showed increased expression of NOS2, NOX1, HO-1, and SOD2. The levels of NOS2 and NOX1 positively correlated with pro-inflammatory cytokines (IL-5, IL-6). In vitro and ex vivo treatments with crocin inhibited macrophage differentiation, decreased NOS2 and NOX1 expression, and activated the antioxidant KEAP1/NRF2/HO-1 signaling pathway. Conclusion: Oxidative stress amplifies inflammatory responses in CRSwNP, and antioxidant compounds such as crocin have therapeutic potential.
Nishida et al., 2020 [15]	Human; case–control molecular pathology	CRSwNP (*n* = 31), CRSsNP (*n* = 13), controls (*n* = 19) ethmoid mucosa and blood	RT-PCR, ELISA, immunostaining for LOX-1 and SR-B1; correlation with CT (Lund–Mackay)	LOX-1 (oxidized LDL receptor) and SR-B1; oxidative stress linkage	*LOX-1* expression was significantly greater in CRSwNP than control tissues, but *SR-B1* expression had no significant differences among control, CRSsNP, and CRSwNP tissues. *LOX-1* was positively correlated with the CT severity score (r = 0.411, *p* < 0.0001) and sinus tissue (but not serum) LOX-1 protein levels were higher in CRSwNP than in controls (*p* < 0.05). Conclusion: LOX-1 (a receptor for oxidized LDL produced by oxidative stress) was upregulated in CRSwNP and associated with disease severity, suggesting it has a role in the onset and chronicity of CRSwNP.
Jiang et al., 2024 [16]	Human scRNA-seq reanalysis + in vitro mechanistic validation	Public scRNA-seq (HRA000772) epithelial cells; nasal mucosa tissues; epithelial cell models with LPS ± MCC950; NOX2 siRNA/plasmid; TMAO	Gene set enrichment for oxidative stress/NLRP3 scores; ROS probes; IHC; inflammasome modulation (MCC950 inhibitor; TMAO inducer)	NOX2-derived ROS; NLRP3 inflammasome; epithelial alarmins	Single-cell RNA-seq identified a higher oxidative stress score and NLRP3 inflammasome score in CRSwNP epithelial cells, and increased epithelial ROS in CRSwNP tissues. The in vitro knockdown of *NOX2* decreased the induction of ROS, NLRP3 inflammasomes, and epithelial alarmins by LPS (IL-33, TSLP) and these effects were significant per figure thresholds (*p* < 0.05 to *p* < 0.0001); TMAO reversed the alarmin decrease, and MCC950 blunted NOX2-driven alarmin upregulation. Conclusion: Epithelial NOX2-driven production of ROS activated NLRP3 inflammasomes, and this led to increased epithelial alarmins and nasal mucosal inflammation in CRSwNP. Therapeutic targeting of NOX2 may inhibit chronic inflammation in CRSwNP.
Zheng et al., 2020 [17]	Human; case–control tissue expression study	CRSwNP (*n* = 13) NP tissue vs. controls (*n* = 9) nasal mucosa	Western blot and qPCR for NOX subunits; IHC/IF for p67phox and 4-HNE	NADPH oxidase subunits (p67phox, p47phox, gp91phox, p22phox); 4-HNE	In nasal polyp (NP) tissues from CRSwNP had greater expression of p67phox than control mucosa based on Western blotting (*p* = 0.004) and real-time PCR (*p* = 0.001). 4-HNE expression (IHC staining) was also significantly higher in NP tissue than control mucosa (*p* = 0.001). p67phox was localized to eosinophils and neutrophils (not macrophages) in NP tissue. Conclusion: NP tissues had higher levels of *p67phox* mRNA and p67phox protein and of an oxidative-stress marker (4-HNE), supporting the role of p67phox and oxidative stress in the pathogenesis of CRSwNP.
Yoon et al., 2020 [18]	Human tissue + in vitro epithelial stimulation	CRSwNP patients (UT and NP) vs. healthy controls; nasal epithelial cells ± SEB stimulation	mtROS measurement; OXPHOS complex assessment; mitochondrial morphology (TEM); fission/fusion markers; MitoTEMPO scavenging	Mitochondrial ROS; SEB; mitochondrial dynamics proteins (Drp1/Fis1, MFN1/OPA1, etc.)	SEB-stimulated nasal epithelial cells had increased mtROS, and MitoTEMPO treatment decreased this effect (*p* < 0.05, *p* < 0.01). SEB also increased the OCR, and MitoTEMPO decreased this effect (*p* < 0.05; *p* < 0.01; *p* < 0.005; § *p* < 0.001). Expression of the oxidative phosphorylation complex and mtROS/Mn-SOD were higher in NP than control UTs. Increased fusion/fission-related molecules and elevated PINK1 in NP versus control UT (*p* < 0.05). Conclusion: Increased production of mtROS, disrupted mitochondrial function, and structural mitochondrial changes in nasal epithelial cells appears to contribute to the pathogenesis of CRSwNP.
Lee et al., 2023 [19]	Human secretions + in vitro ALI epithelial infection model	CRSsNP (*n* = 23), CRSwNP (*n* = 26), controls; normal sinonasal epithelial cells infected with RV16 or poly(I:C) ± H2O2/NAC	H2O2 in nasal secretions; RT-qPCR/ELISA/WB for IFN-β, IFN-λ1/2 and ISGs; PRR signaling (TLR3, RIG-I, MDA5, IRF3); Nrf2 siRNA and sulforaphane	Oxidative stressor H2O2; antioxidant NAC; Nrf2 pathway; antiviral interferon response	Nasal lavage H_2_O_2_ levels were higher in CRS than in controls, and the highest levels were in CRSwNP, and in ALI-cultured normal sinonasal epithelial cells. Application of RV16 infection or poly I:C increased type I/III interferons and ISGs, but this upregulation was attenuated by H_2_O_2_ pretreatment (*p* < 0.05). The suppressive effects of H_2_O_2_ on interferons, ISGs, and PRR signaling did not occur after NAC pretreatment. Nrf2 knockdown decreased (and sulforaphane increased) the antiviral interferon responses after RV16 or poly I:C. Conclusion: Oxidative stress (modeled by H_2_O_2_) attenuated RV16-induced antiviral production of interferons in sinonasal epithelial cells, and this was associated with downregulation of PRR/IRF3-related signaling.
Shin et al., 2019 [20]	Human primary nasal epithelial ALI model	Inferior turbinate epithelial cells from septal surgery patients (*n* = 10)	Intracellular ROS measurement; transepithelial resistance (TER); RT-PCR/Western/confocal for tight junction proteins; protease inhibition/heat inactivation	Alternaria serine protease; ROS; tight junction proteins (ZO-1, occludin, claudin-1)	*Alternaria* increased intracellular ROS and decreased TER, and these responses did not occur when *Alternaria* was heat-inactivated or when tissues or *Alternaria* were treated with glutathione or a serine protease inhibitor (Pefabloc, *p* < 0.05). *Alternaria* also decreased tight-junction molecules (ZO-1, occludin, claudin-1) at the mRNA and protein levels (*p* < 0.05) but did not affect an adherens-junction molecule (E-cadherin). Conclusion: Serine protease in *Alternaria* disrupted nasal epithelial barrier function, and its induction of intracellular ROS may have contributed to this impairment and promoted inflammation of the nasal mucosa.
Zhang et al., 2025 [21]	Human scRNA-seq/tissue + mouse CRS model + ALI epithelial cultures	Human CRSwNP vs. controls; CRS mouse model; human nasal epithelial ALI cultures; APE1 redox inhibitor (C10)	scRNA-seq, IHC/WB/qRT-PCR; in vivo C10 treatment; transcriptomics; ROS/mitochondrial damage assays; barrier and cytokine outcomes	APE1/Ref-1 redox activity; ROS/mitochondrial stress; type 2 cytokines and IL-25 (tuft cells)	Polyp tissue from patients with CRSwNP indicated a positive correlation of APE1 expression with eosinophils (R^2^ = 0.6891, *p* = 0.0008), MUC5AC (R^2^ = 0.7384, *p* = 0.0003), and ROS (R^2^ = 0.6117, *p* = 0.0026), and negative correlations with ZO-1 (R^2^ = −0.8355, *p* < 0.0001) and antioxidative genes including SOD2 (R^2^ = −0.8526, *p* < 0.0001), NRF2 (R^2^ = −0.7151, *p* = 0.0005), FOXO1 (R^2^ = −0.826, *p* < 0.0001), and TXN2 (R^2^ = −0.8978, *p* < 0.0001). Conclusion: Mouse and epithelial models indicated that C10-mediated inhibition of APE1 redox function decreased tuft-cell IL-25/type 2 inflammation and mitigated ROS/mitochondrial oxidative stress, suggesting that targeting of APE1 redox function has therapeutic potential in CRS.
Gao et al., 2026 [22]	Human tissue proteomics + in vitro mechanistic study	CRSwNP NP tissues vs. healthy controls; cultured nasal epithelial cells with H2O2 ± PRDX2 overexpression	Label-free proteomics; ROS staining; IF and RT-PCR validation; EMT markers; TGF-β1/SMAD signaling assays	PRDX2 (antioxidant enzyme); ROS; EMT/remodeling markers (E-cadherin, Vimentin) and TGF-β1/SMAD pathway	Polyp tissue from patients with CRSwNP had a greater level of ROS than those with eCRSwNP (eCRSwNP vs. neCRSwNP, *p* < 0.0001). PRDX2 as the most significantly downregulated protein in CRSwNP (confirmed by RT-PCR and IF; *p* < 0.01, *p* < 0.0001). In NECs, H_2_O_2_ induced ROS and the EMT, but PRDX2 overexpression mitigated the induction of ROS and the EMT and suppressed TGF-β1/Smad2/3 activation (*p* < 0.05). Conclusion: ROS accumulation is critical in CRSwNP. PRDX2 regulated ROS-induced epithelial remodeling via the TGF-β1/SMAD pathway, thereby altering disease progression.
Wu et al., 2019 [23]	Human primary fibroblast in vitro study	Nasal polyp–derived fibroblasts vs. normal mucosa fibroblasts; bleomycin A5 ± glutathione	DCFH-DA ROS probe; apoptosis assays (Annexin V/PI, TUNEL); migration/cell cycle; Western blot for apoptosis and ECM proteins	Mitochondrial ROS; glutathione (GSH); ECM proteins (collagen/aggrecan) and apoptosis markers	NPDFs had a higher level of basal ROS and more active growth than NMFCs, and NPDFs were more sensitive to BLE-5A-induced apoptosis and had greater production of ROS. GSH abrogated the BLE-5A-induced degradation of ECM and apoptosis in NPDFs. These effects occurred *via* a mitochondrial-mediated pathway. Conclusion: BLE-A5 induced the apoptosis of NPDFs and ECM degradation via a mitochondrial-mediated and ROS-dependent mechanism.
Fordham et al., 2013 [24]	Human; retrospective cohort	69 adults undergoing sinus surgery; controls vs. CRSsNP vs. CRSwNP; stratified by SHS exposure (hair nicotine)	DAB staining in sinus tissue to quantify ROS-positive cells; hair nicotine for SHS exposure	Tissue ROS burden (DAB-positive cells)	In cohorts not exposed to SHS, there were more DAB-positive cells/hpf in CRS patients than in controls (CRSsNP: median 1.44, 0.17–4.00, *p* = 0.0049; CRSwNP: median 10.77, 6.4–14.5, *p* = 0.0006), and CRSwNP had more DAB-positive cells/hpf than CRSsNP (*p* < 0.0001). SHS exposure increased DAB-positive cells/hpf in controls (0 vs. 0.56, 0.11–0.65; *p* = 0.01), but not in CRSsNP (*p* = 0.21) or CRSwNP (*p* = 0.88). Conclusion: ROS occurred at different levels in different CRS subtypes, and SHS only increased the ROS in the sinus tissue of controls, with uncertain clinical significance.
Tsai et al., 2025 [25]	Human; PCR-array transcriptomics + validation	Control vs. CRSsNP nasal mucosae	84-gene redox PCR array; RT-PCR/WB validation; IHC for 4-HNE and 3-nitrotyrosine; network analysis comparing CRSsNP vs. CRSwNP	OxS/NsS markers; redox DEGs (e.g., AKR1C2, GCLM, GPX2, NOS2, NQO1 up; LPO down)	An 84-gene PCR array of redox related genes indicated 27 DEGs in CRSsNP relative to controls (24 upregulated, 3 downregulated), with key increases in *NOS2* and *NQO1* and a decrease in *LPO*. IHC showed increased oxidative/nitrosative damage in CRSsNP mucosa based on increased 4-HNE and 3-nitrotyrosine staining (*p* < 0.01 and *p* < 0.0001 vs. control). Conclusion: Multiple DEGs were associated with increased oxidative stress in CRSsNP nasal mucosa. Network analysis suggested CRSsNP had an ‘adaptive antioxidant defense’ signature, whereas CRSwNP had a ‘pro-inflammatory- and -oxidant pathway’ signature.
Lawrence et al., 2015 [26]	Human ex vivo primary cell culture	HSNECs from controls (*n* = 7) and CRSwNP (*n* = 9); exposed to Aspergillus/Alternaria ± SOD	Epithelial culture; cytokines IL-6 and IL-8 by ELISA after fungal antigen exposure	Superoxide dismutase (SOD) as ROS scavenger; IL-6/IL-8 output	In HSNECs from control and CRSwNP patients, *Aspergillus* and *Alternaria* increased the production of IL-6 and IL-8 (*p* < 0.05). Co-treatment with SOD decreased this response in CRSwNP HSNECs (significant for IL-6 and IL-8 after *Alternaria*, and for IL-8 after *Aspergillus*, *p* < 0.05), but not in control HSNECs. Conclusion: SOD decreased fungal antigen–induced inflammation in CRSwNP-derived HSNECs based on measurements of IL-6 and IL-8.
Kohanski et al., 2016 [27]	Human in vitro ALI culture study	13 control subjects (no CRS) for ALI culture; ROS assay performed using SNECs from 13 patients; ELISA/LDH assays reported for 5 patients.	ALI culture; DCFDA fluorescence for ROS; RT-PCR for Nrf2-regulated genes and inflammatory genes; ELISA (TNF-α, IL-1β); LDH secretion for cytotoxicity.	ROS (DCFDA); bactericidal antibiotics (levofloxacin, amoxicillin) vs. bacteriostatic (clarithromycin); Nrf2-mediated antioxidant genes (GCLC, GCLM, NQO1, p62, etc.); inflammatory mediators (TNF-α, IL-1β, IL-18, CASP1); cytotoxicity (LDH).	Levofloxacin and amoxicillin increased production of ROS (*p* = 0.013, *p* = 0.008), but clarithromycin did not. These two bactericidal antibiotics also increased antioxidant/inflammatory responses (TNF-α: levofloxacin *p* = 0.037, amoxicillin *p* = 0.002) and the levels of inflammasome-related genes including IL-1β (*p* = 0.0002) and LDH at 48 and 96 h (*p* < 0.05). Conclusion: Bactericidal antibiotics increased the formation of ROS, the expression of inflammatory- and antioxidant-related genes, and cell death. Oxidative damage of the epithelium may occur following long-term or inappropriate use of certain bactericidal antibiotics.
Kohanski et al., 2017 [28]	Human; in vitro ALI-cultured SNECs treated with levofloxacin ± sulforaphane (Nrf2 activator) to probe ROS–apoptosis linkage.	10 subjects total; 7 controls and 3 CRS (tissue from ethmoid sinuses during ESS).	DCFDA fluorescence for ROS; caspase-3 colorimetric assay; TUNEL for DNA fragmentation; sulforaphane pretreatment experiments.	ROS (DCFDA); apoptosis markers caspase-3 activity, DNA fragmentation (TUNEL); antioxidant pathway modulation via sulforaphane/Nrf2.	Levofloxacin increased ROS at 40 and 80 μg/mL after 24 h and 72 h (*p* < 0.05) and increased the levels of apoptosis markers, including caspase-3 (5.9-fold, *p* = 0.01) and TUNEL-positive cells. Sulforaphane (80 μg/mL) decreased the apoptotic response (TUNEL assay: *p* = 0.02). Conclusion: Levofloxacin triggered ROS-associated programmed cell death in SNECs, and sulforaphane suppressed this effect. Long-term or inappropriate antibiotic exposure may lead to oxidative damage of tissues in the sinonasal epithelium.

Abbreviations: AKR1C2, aldo-keto reductase family 1 member C2; ALI, air–liquid interface; APE1, apurinic/apyrimidinic endonuclease 1; ASC, apoptosis-associated speck-like protein containing a CARD; ATP, adenosine triphosphate; AUC, area under the curve; BLE, bleomycin A5; CASP1, caspase 1; CI, confidence interval; CRS, chronic rhinosinusitis; CRSsNP, chronic rhinosinusitis without nasal polyps; CRSwNP, chronic rhinosinusitis with nasal polyps; CT, computed tomography; DAB, diaminobenzidine; DCFDA, 2′,7′-dichlorofluorescein diacetate; DCFH-DA, 2′,7′-dichlorodihydrofluorescein diacetate; DEGs, differentially expressed genes; DNA, deoxyribonucleic acid; Drp1, dynamin-related protein 1; DUOX, dual oxidase; DUOX1, dual oxidase 1; DUOX2, dual oxidase 2; ECM, extracellular matrix; ELISA, enzyme-linked immunosorbent assay; EMT, epithelial–mesenchymal transition; ESS, endoscopic sinus surgery; EthD-I, ethidium homodimer-1; FESS, functional endoscopic sinus surgery; FISH, fluorescence in situ hybridization; FOXO1, forkhead box O1; GCLC, glutamate–cysteine ligase catalytic subunit; GCLM, glutamate–cysteine ligase modifier subunit; GPX2, glutathione peroxidase 2; GSH, glutathione; GSDMD-NT, gasdermin D N-terminal fragment; H_2_O_2_, hydrogen peroxide; H_2_DCFDA, 2′,7′-dichlorodihydrofluorescein diacetate; HMOX1, heme oxygenase 1; HNECs, human nasal epithelial cells; HNEpCs, human nasal epithelial progenitor cells; HO-1, heme oxygenase 1; HSNECs, human sinonasal epithelial cells; IF, immunofluorescence; IFN-β, interferon beta; IFN-λ1, interferon lambda 1; IHC, immunohistochemistry; IL-1β, interleukin 1 beta; IL-6, interleukin 6; IL-8, interleukin 8; IL-18, interleukin 18; IL-25, interleukin 25; IL-33, interleukin 33; IRF3, interferon regulatory factor 3; ISGs, interferon-stimulated genes; JC-1, 5,5′,6,6′-tetrachloro-1,1′,3,3′-tetraethylbenzimidazolylcarbocyanine iodide; KEAP1, Kelch-like ECH-associated protein 1; LDH, lactate dehydrogenase; LDL, low-density lipoprotein; LOX-1, lectin-like oxidized low-density lipoprotein receptor 1; LPO, lactoperoxidase; LPS, lipopolysaccharide; MCC950, NLRP3 inflammasome inhibitor MCC950; MDA, malondialdehyde; MDA5, melanoma differentiation-associated protein 5; MFN1, mitofusin 1; MIG, monokine induced by interferon-γ; ML385, Nrf2 inhibitor ML385; MMP, matrix metalloproteinase; MPO, myeloperoxidase; mRNA, messenger RNA; mtROS, mitochondrial reactive oxygen species; MUC5AC, mucin 5AC; NAC, N-acetylcysteine; NADPH, nicotinamide adenine dinucleotide phosphate; NCF2, neutrophil cytosolic factor 2; NECs, nasal epithelial cells; neCRSwNP, non-eosinophilic chronic rhinosinusitis with nasal polyps; NLRP3, NOD-like receptor family pyrin domain-containing 3; NMFCs, normal mucosa-derived fibroblasts; NO, nitric oxide; NOS2, nitric oxide synthase 2; NOX, NADPH oxidase; NOX1, NADPH oxidase 1; NOX2, NADPH oxidase 2; NP, nasal polyp; NPDFs, nasal polyp-derived fibroblasts; NQO1, NAD(P)H quinone dehydrogenase 1; NRF2, nuclear factor erythroid 2-related factor 2; OCR, oxygen consumption rate; OPA1, optic atrophy protein 1; OXPHOS, oxidative phosphorylation; OXR1, oxidation resistance 1; PCR, polymerase chain reaction; PINK1, PTEN-induced kinase 1; PLD, platycodon D; PRDX2, peroxiredoxin 2; PRDX6, peroxiredoxin 6; PRNP, prion protein; PRR, pattern recognition receptor; qPCR, quantitative polymerase chain reaction; qRT-PCR, quantitative reverse transcription polymerase chain reaction; Ref-1, redox effector factor 1; RIG-I, retinoic acid-inducible gene I; RNA, ribonucleic acid; ROS, reactive oxygen species; RT-PCR, reverse transcription polymerase chain reaction; RT-qPCR, reverse transcription quantitative polymerase chain reaction; RV16, rhinovirus 16; scRNA-seq, single-cell RNA sequencing; SEB, *Staphylococcus aureus* enterotoxin B; SHS, secondhand smoke; siRNA, small interfering RNA; SMAD, suppressor of mothers against decapentaplegic; SNECs, sinonasal epithelial cells; SOD, superoxide dismutase; SOD2, superoxide dismutase 2; SOD3, superoxide dismutase 3; SR-B1, scavenger receptor class B type 1; TEM, transmission electron microscopy; TER, transepithelial resistance; TGF-β1, transforming growth factor beta 1; TLR3, toll-like receptor 3; TMAO, trimethylamine N-oxide; TNF-α, tumor necrosis factor alpha; TRX, thioredoxin; TSLP, thymic stromal lymphopoietin; TUNEL, terminal deoxynucleotidyl transferase dUTP nick end labeling; TXN2, thioredoxin 2; TXNIP, thioredoxin-interacting protein; UT, uncinate tissue; WB, Western blot; ZO-1, zonula occludens 1.

## Data Availability

No new data were created or analyzed in this study. Data sharing is not applicable to this article.
